# Increased microsaccade rate in individuals with ADHD traits

**DOI:** 10.16910/jemr.10.1.6

**Published:** 2017-03-04

**Authors:** Maria Panagiotidi, Overton Paul, Stafford Tom

**Affiliations:** Staffordshire University, Stoke-on-Trent, UK; The University of Sheffield, UK

**Keywords:** eye movement, eye tracking, microsaccades, ADHD, eye movements, superior colliculus

## Abstract

Microsaccades are involuntary, small, jerk-like eye-movements with high-velocity that are observed during fixation. Abnormal microsaccade rates and characteristics have been observed in a number of psychiatric and developmental disorders. In this study, we examine microsaccade differences in 43 non-clinical participants with high and low levels of ADHDlike traits, assessed with the Adult ADHD Self-Report Scale [28]. A simple sustained attention paradigm, which has been previously shown to elicit microsaccades, was employed. A positive correlation was found between ADHD-like traits and microsaccade rates. No other differences in microsaccade properties were observed. The relationship between ADHD traits and microsaccades suggests that oculomotor behaviour could potentially lead to the development of a biomarker for the ADHD.

## Introduction

Our ability to see depends partly on being able to align our eyes with a visual target. Eye-movement behaviour is highly optimized to satisfy these needs; most of the time our eyes scan visual scenes in sequences of saccades and fixations. Saccades are rapid, ballistic eye movements that bring the fovea to specific portions of the visual field. Fixations, on the other hand, maintain the visual gaze to a specific location keeping a target relatively stable with respect to the retina. Even though the definition of a fixation might suggest that the eyes remain stable, in reality the eyes are moving continuously. More specifically, when fixating on a stationary object our eyes perform tiny, seemingly erratic fixational eye movements. These eye movements are not performed voluntarily and we are generally not aware of their existence.

The most prominent contribution to fixational eye movements is generated by small (a few arc min to 1.0º), jerk-like eye-movements with high-velocity that are embedded into slower drifting movements [33]. On average an individual executes about 1-2 microsaccades per second [7]. These eye movements were first discovered by Dodge [8] and are known as microsaccades, a term introduced by Zuber, Crider, and Stark [58]. Before the term microsaccades was introduced and adopted in vision studies, they could be found in the literature with various names such as small, miniature, or fixational saccades, mini-saccades, jerks, flicks, jumps [48].

Animal studies have found that the superior colliculus (SC), a multimodal laminar structure located in the midbrain that belongs to a distributed network of areas mediating saccadic eye movements, and shifts of attention, plays a key role in the microsaccade generation [23, 20]. Hafed [23] investigated the temporal profile of microsaccade-related activity in the SC. They found that microsaccade-related activity in the SC tends to increase gradually before the execution of a microsaccade, peaks around the time the eye movement begins, and gradually returns to baseline levels after the execution of the microsaccade. Goffart and colleagues [15] showed a direct connection between the SC and microsaccades executed during sustained fixation periods. After deactivating the rostral deep SC of monkeys they observed a significant drop in the microsaccade rate during fixation. Furthermore, reversible inactivation of the SC has been shown to disrupt the ability of peripheral cues to bias the direction of microsaccades [24]

The SC has recently garnered interest as one potential site of dysfunction in Attention Deficit Hyperactivity Disorder [41, 6], the most prevalent neurodevelopmental disorder [52]. It is proposed that the colliculus is hyperresponsive to sensory stimuli in patients with ADHD [41, 6]. These patients show increased distractibility in tasks that are sensitive to collicular function, and have a general problem inhibiting saccades, the generation of which involves the SC [41, 6]. Furthermore, damphetamine, a drug used therapeutically in ADHD, depresses collicular responses to visual stimuli [17]. As a result, examining the microsaccades in ADHD could provide further evidence for or against collicular involvement in the disorder and, if the former, could play a useful role diagnostically in the future. However, only a very small number of studies have examined microsaccades in patient groups.

Variations in microsaccade rates across participants are a common finding in studies examining eye movements [26, 12]. A study by Poynter, Barber, Inman, & Wiggins [46] who examined individual differences in eyemovement behaviour in a 40 subjects, found that normal individuals who reported relatively high levels of attention problems exhibited relatively frequent fixations of short duration and large spatial extent. The metric they developed measured the extent of all types of fixational eye-movements (i.e. tremors, drifts), not microsaccades exclusively.

A number of previous studies have reported gaze instability and increased eye movements in children and adults with ADHD [2, 16, 37, 31]. Only one study so far has investigated microsaccades in ADHD. Fried and colleagues [13] found that adults with ADHD off medication make more microsaccades compared to a group of controls when engaged with a continuous performance task. The difference between groups was pronounced in peri-stimulus trials; when the participant was anticipating a target and was required to suppress eye-movements. With medication (methylphenidate) the microsaccade rate in the ADHD group was normalized, suggesting a potential relationship between ADHD medication and microsaccade generation.

However, the disadvantage of working with a clinical ADHD population is that the majority of individuals with ADHD have been treated chronically with psychostimulant medication (the front-line treatment for ADHD; [3]), which is known to affect oculomotor function [9]. Fortunately, ADHD psychopathology can be viewed dimensionally, with inattentive and hyperactive-impulsive symptoms distributed continuously in the general population [27]. Preliminary evidence suggests that ADHD represents the extreme end of traits present in the general population [32]. Hence, here we examined differences in microsaccade rates and microsaccade characteristics in non-clinical participants with varying levels of ADHD-like traits. The dimensional approach has been widely used in studies on other developmental disorders such as autism spectrum disorder (ASD) (Dickinson et al., 2014) and has been recently employed by researchers investigating ADHD [45].

## Methods

### Participants

43 participants (35 female) were recruited from the volunteers’ list of the University of Sheffield. The ages of the participants varied from 18 to 30 (M = 20.72, SD = 3.33). All subjects had normal or corrected-to-normal vision and were naïve as to the purpose of the experiment. Four participants were left-handed. All the participants were healthy and none were previously diagnosed with ADHD or any other major mental illness.

They were all awarded for their time with credits needed for the completion of their undergraduate degree. The subjects all gave their informed consent to take part in the experiment and the procedures were conducted in accordance with the Code of Ethics of the World Medical Association (Declaration of Helsinki).

### Materials and Procedure

The Adult ADHD Self-Report Scale [28] was administered to determine ADHD-like traits in participants. The ASRS is an instrument consisting of the 18 DSM-IV-TR criteria and was developed in conjunction with the World Health Organization (WHO), and the Workgroup on Adult ADHD. The scores obtained through the ASRS have been found to be predictive of symptoms consistent with ADHD [1]. The ASRS contains eighteen items from DSM-IV-TR (American Psychiatric Association, 2000) but measures the frequencies of the symptoms. The subjects are asked to report how often they experience each symptom in a period of six months on a five point Likert scale which ranges from 0 for never, 1 for rarely, 2 for sometimes, 3 for often, and 4 for very often [28]. The ASRS has a two factor structure [47] which includes an inattention scale and a hyperactivity/impulsivity scale. Each subscale contains nine items. The ASRS examines only current adult symptoms of ADHD. The reliabilities (Cronbach’s alpha) for the two subscales of inattention (.75) and impulsivity (.77) as well as for the total ASRS (.82) are satisfactory [47]. The original questionnaires are formatted with darkly shaded boxes in certain items which signify more severe symptoms, but these were removed from the questionnaire administered to our participants to avoid potential bias in the responses.

The participant was seated in front of an the Eyelink 1000 video-based eye tracker (SR Research Osgood, ON, Canada), in a padded chair located about 100 cm away from the screen. The eyetracker was mounted on a headand-chin rest. Horizontal and vertical eye positions for both eyes were sampled at a rate of 500 Hz (pupil-only mode, instrument noise 0.01 deg RMS) and stored for offline analysis. A keyboard was positioned within easy reach in front of the subject. Subjects were asked to maintain a stable posture and head position during the course of the experiment.

Before the main task begun, calibration and validation were performed. Calibration was manual and based on a number of 9 (grid) points. Participants were required to produce saccades towards 9 fixation points sequentially appearing at random on the screen. After calibration was performed, validation was performed by re-presenting the targets and determining the magnitude of the calibration error. In case the validation was unsuccessful, calibration was repeated. The process took approximately 2 min for each participant. Drift correction was performed before every trial. During the drift correction, the participant was presented with a blank screen and the same black marker presented in the calibration and they were instructed to focus on the black marker fixation point. Calibration was performed twice for each participant; at the beginning of each block.

**Figure 1:**
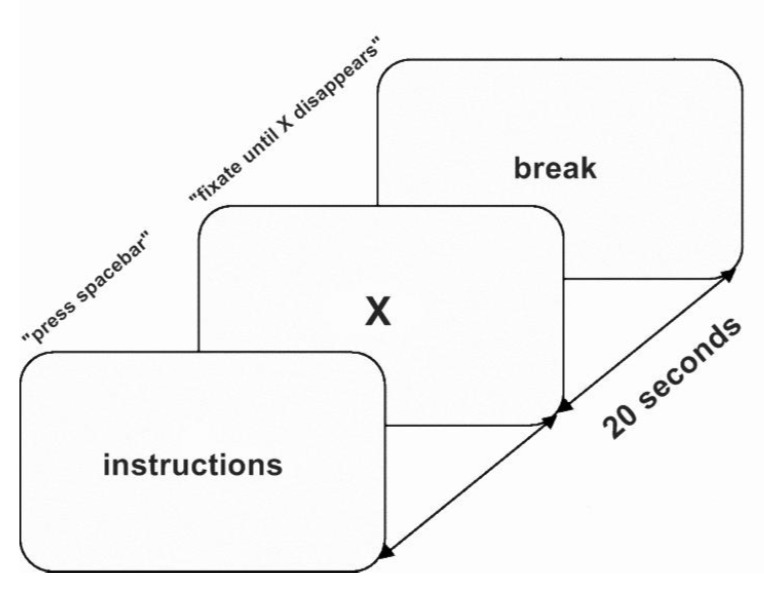
Graphical representation of the sustained fixation task: Participants were instructed to fixate on a white cross appearing on a black screen for 20 seconds. Each trial was followed by a short break.

Once the calibration and validation were successfully performed, the main task was administered. It consisted of a simple sustained fixation task (Figure 1). Participants were instructed to fixate on a white cross appearing on a black background in the centre of 20 inch Mitsubishi Diamondpro 2070sb (86 Hz refresh rate) screen for 20 seconds.

Overall, 2 blocks of 10 trials were presented to each participant. There was a break between each trial; the experiment was self-paced and the participant was asked to press the space-bar to begin each trial. Each trial was preceded by the instructions screen. The stimuli were presented with OpenSesame [34] using the PsychoPy [44] back-end. Each trial lasted for 20 seconds.

### Data Analysis

**Figure 2:**
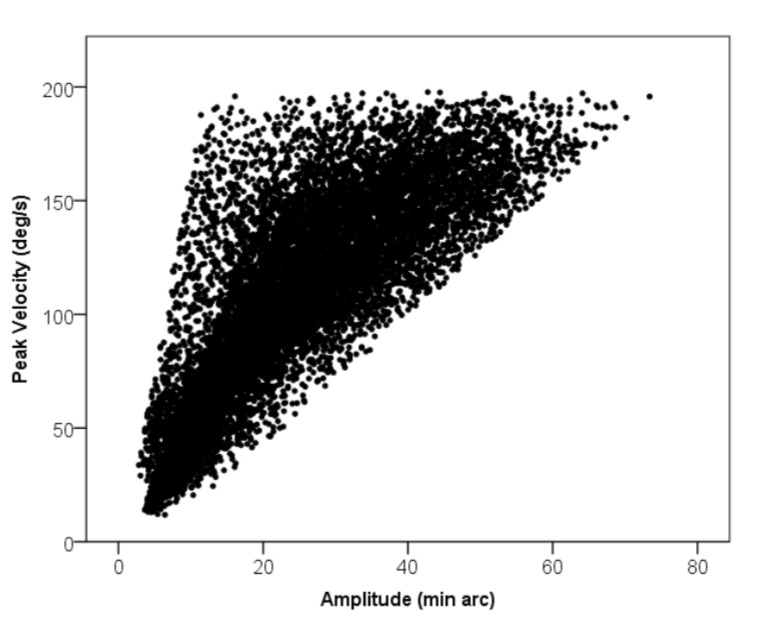
The peak microsaccade velocities were plotted against their amplitudes. N = 12940

Microsaccades were detected using the algorithm of Engbert and Kliegl [11] adapted to a 500-Hz sampling rate. This particular algorithm is one of the most commonly used in microsaccade research and has been found reliable in detecting microsaccades [11, 10, 40]. The algorithm developed by Engbert and Kliegl [12] allows the detection of binocular microsaccades (i.e., eye movements that occur in both eyes at the same time and at least one sample overlaps in time) and monocular microsaccades (i.e., movements that occur in one eye). The average horizontal and vertical displacement across the two eyes was used to determine the amplitude and direction of binocular microsaccades [12, 10, 40]. Samples where no tracking data were detected were characterized as blinks and were excluded from the microsaccade analysis. Furthermore, to prevent false detection of microsaccades around blinks, an additional 20 ms before and after each blink was excluded. This was done to avoid noise induced to data by blinks [53]. We used a value λ=6 in all computations reported here. A minimal duration of three data samples (12 ms) was assumed in order to further reduce noise. The characteristics of the detected movements were manually checked and confirmed by plotting peak microsaccade velocity against amplitude (Figure 2), as well as by plotting amplitude distributions [58, 23, 19].

All the trials from all the sessions were collapsed and included in the analysis. First, the position data was transformed to velocities using a moving average of 3 data samples (6 ms) for each eye. Second, the velocity threshold was computed and then multiplied by the relative velocity threshold (6.0). If the average velocity exceeded the velocity threshold in at least three consequent samples, the movement was defined as monocular microsaccade. The microsaccades extracted from the algorithm showed a strong correlation between peak velocity and amplitude (r = 0.81, p <.01 (2-tailed)). These results are consistent with the standard main sequence effect in the literature [35, 13]. Microsaccades with amplitudes exceeding 2º and velocities over 200 were excluded from further analysis as in Yokoyama, Noguchi, & Kita [56]. Only binocular microsaccades were included in the analysis.

Data from three participants were excluded from the analysis as they failed to complete all 20 trials. Another participant was excluded from the analysis due to high levels of noise in the data (e.g. the algorithm detected an unusual number of microsaccades (over 10 microsaccades /sec) in the sample). As a result, data from 38 participants were included in the final analysis. The mean proportion of excluded data from the analysis due to the issues described above was 16.8% (SD=15.7). A minimum of 16 trials were included in the analysis for each participant. No relationship was found between number of excluded trials and ADHD traits (r(38)=.11, p=.47)

## Results

### ASRS Scores

Scores on the ASRS checklist varied from 23 to 58 and the mean score was 35.74 (SD= 9.6). The mean score on the inattention subscale was 20.29 (SD= 4.8) and the hyperactivity subscale 15.45 (SD= 6.39). The two subscales were correlated, r(38)=.459, p<.01. The overall ADHD score was correlated with both the inattention (r(38)=.807, p<.01) and the hyperactivity subscale (r(38)=.896, p<.01). Since the two subscales were strongly correlated, only overall ASRS scores were used in the analysis.

### Microsaccade Features

Microsaccades have often been shown to follow the main sequence; an approximately linear relation between the amplitude of the eye movement and the peak velocity. The peak microsaccade velocities were plotted against their amplitudes in our data. A significant linear relationship was observed (Figure 2). The mean microsaccade rate was .81 microsaccades per second (SD = .48, Min = .1, Max = 2.04).

### Relationship between ADHD Traits and Microsaccades

**Figure 3:**
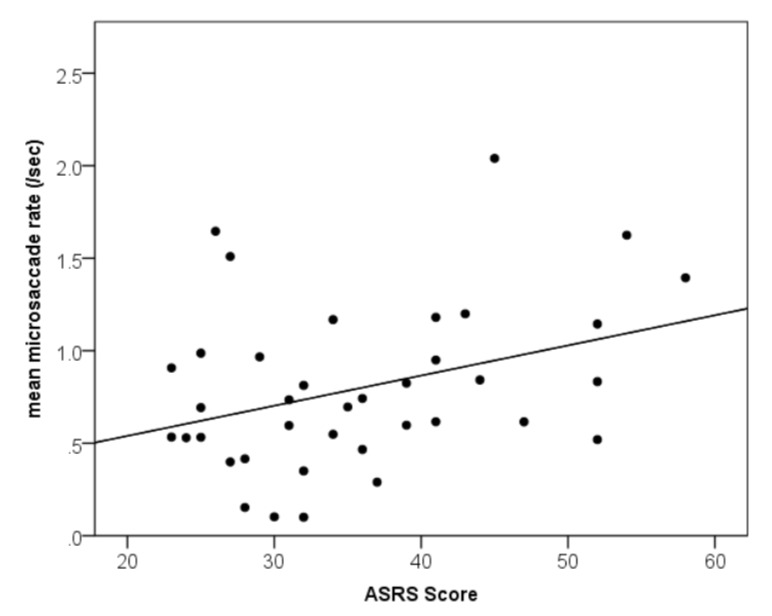
Relationship between ADHD traits and mean binocular microsaccade rate (microsaccades/sec).

A positive correlation was found between overall ASRS scores and binocular microsaccade rate (Figure 3), r(38)=.35, p=.02. Higher level of ADHD traits was associated with an increased rate microsaccades.

No correlation was found between ASRS scores and mean microsaccade peak velocity, r(38)=-.04, p=.821. There was also no correlation between ADHD traits and microsaccade amplitudes, r(38)=.213, p=.21.

### ADHD Traits and Tracking Noise

A way to investigate tracking noise in the detection of microsaccades involves examining the relationship between saccade amplitude and peak velocity, known as the main sequence [58, 25]. No correlation was found between the slope of the linear fit of the main sequence and ADHD traits (r(38) = -.043, p =.8).

## Discussion

This study examined microsaccade rates in nonclinical participants with varying levels of ADHD-like traits during a prolonged sustained fixation paradigm. We found that a higher level of ADHD-like traits as measured in the ASRS was associated with increased rates of microsaccades. Participants who reported more inattention and hyperactivity traits made more microsaccades. This is the second study showing a connection between ADHD symptoms and microsaccade rate [13], and extends the findings of the former study to a drug free adult population. The finding suggests that abnormal fixational eye movements could be part of the ADHD phenotype. The SC plays an important role in the generation and inhibition of microsaccades [23]. If the SC is hyper-responsive in individuals with ADHD as the SC dysfunction hypothesis suggests [41], it might be expected that this would lead to an overproduction of microsaccades and difficulty sustaining fixation. As a result, our findings offer support to the idea that the SC is dysfunctional in ADHD.

A deficit in top-down regulation could also explain the relationship between ADHD traits and microsaccade frequency. A number of ADHD symptoms such as distractibility and inattention have been attributed to deficits in executive functions and the frontal lobes (Barkley, 1997). In particular, regions such as the frontal eye fields have been shown to regulate microsaccade generation [43]. As a result, increased microsaccade rates could be due to deficits in top-down modulation.

Deficits in response inhibition when fixating on a static target could also lead to an increased rate of microsaccades. Response inhibition has been identified as one of the core deficits of ADHD (Barkley, 1999). Studies have demonstrated that children with ADHD have difficulty inhibiting automatic responses and carrying out goaloriented behaviour [31].

Our paradigm employed trials with fixed timing. It has been shown that fixed timing is a critical property and that people with ADHD have a specific impairment in the transient allocation of attention for anticipated and regular events [51, 13]. Therefore, it is possible that participants with higher ADHD traits found the task more demanding. Previous research has shown that microsaccade rates and amplitudes can be affected by task difficulty [50, 14, 29]. Laubrock and colleagues [29] used variations of a Posner cuing paradigm and found an inverse relationship between microsaccade rate and subjective task difficulty. Pastukhov and Braun [42] examined the effect of attentional load on microsaccade rate during a visual recognition tasks and found that higher cognitive loads were associated with reduced microsaccade rates. Similar findings have been reported by studies employing non-visual tasks. For example, Siegenthaler, Costela, McCamy, Di Stasi, Otero‐ Millan, Sonderegger, Groner, Macknik, & Martinez‐ Conde, [50] used a mental arithmetic task and found that microsaccade rates decreased with increasing difficulty. A similar relationship was reported by Gao and colleagues [14], who employed an auditory task. Even though the majority of previous research suggests a negative relationship between task difficulty and microsaccade rates, there is some conflicting evidence. Benedetto, Pedrotti, & Bridgeman [4] reported increased frequency of microsaccades in a dual-task driving simulator task compared to a simple driving task. Further studies should attempt to investigate the effect of task difficulty on microsaccade rates in individuals with ADHD

Individual measures of eye-movement behaviour have been shown to be consistent across tasks [46]. For example, Poynter and colleagues [46] used six metrics (Fixation Rate, Duration, and Size; Saccade Amplitude; MicroSaccade Rate and Amplitude) to measure individuals’ eye-movement behaviour profiles and observed stable idiosyncrasies in measures of fixational eye-movement and consistent inter-metric correlations across tasks (e.g. participants who make more frequent saccades in one task, executed saccades more frequently in a different task). Similar findings have been reported by Hermens & Walker [26] who found that microsaccade rates within individuals were similar across different conditions. Consequently, ADHD traits could be associated with an increased number of microsaccades in different tasks. Failing to suppress microsaccades could be linked to attention problems. In our study, we used a simple sustained fixation paradigm. Future studies should attempt investigating microsaccades in subjects with ADHD or high ADHD traits in more attention demanding task and correlate their oculomotor pattern with symptom severity.

### A biomarker for ADHD?

ADHD diagnosis is currently based on interviews and parent/guardian/teacher reports. All these measures are characterized by high subjectivity. The subjective nature of ADHD diagnosis hinders ADHD research and its effective treatment. Since there is no objective test for ADHD, other conditions with similar symptoms often get misdiagnosed as ADHD [5]. Several attempts have been made towards the development of an objective test for ADHD. Currently, there is no objective test for ADHD. The majority of developed tests depend on higher cognitive functions, such as sustained and selective attention [18]) and have questionable reliability as screening diagnostic tools [57, 30]. The development of an objective biomarker could lead to more accurate diagnosis and provide an effective way to monitor the effect of treatment [36]. Oculomotor biomarkers are a possible area of interest for ADHD research. In particular, recent studies have found that saccadic abnormalities appear to be capable of distinguishing different diseases and disorders [55].

Here we found a statistically significant correlation between the frequency of microsaccades executed during a sustained fixation task and ADHD traits. Participants with a higher level of ADHD-like traits, as assessed on the ASRS, made more microsaccades while fixating on a target. We acknowledge that the results presented here are preliminary and their diagnostic power at the individual level remains to be further developed and tested. A simple sustained fixation task requires minimum cognitive effort from the participants. In addition to this, eye tracking systems are becoming increasingly popular and their cost is decreasing. It is estimated that in the near future eye tracking will be integrated in gadgets we use on a regular basis. A biomarker based on eye movements could be a very effective way of testing for ADHD traits. Future research should focus on microsaccades.

## Acknowledgements

The authors declare that there is no conflict of interest regarding the publication of this paper.
